# Glymphatic dysfunction as a potential driver of cerebral iron deposition in Parkinson's disease

**DOI:** 10.1093/braincomms/fcaf365

**Published:** 2025-09-23

**Authors:** Kaidong Chen, Yi Ji, Li Zhang, Ruixuan Zhang, Jianqi Li, Liujia Lu, Ying Tang, Xiaoyun Hu, Feng Wang, Xiangming Fang

**Affiliations:** Department of Radiology, The Affiliated Wuxi People's Hospital of Nanjing Medical University, Wuxi 214023, China; Department of Radiology, The Affiliated Wuxi People's Hospital of Nanjing Medical University, Wuxi 214023, China; Department of Neurology, The Affiliated Wuxi People's Hospital of Nanjing Medical University, Wuxi 214023, China; Department of Radiology, The Affiliated Wuxi People's Hospital of Nanjing Medical University, Wuxi 214023, China; Shanghai Key Laboratory of Magnetic Resonance, School of Physics and Electronic Science, East China Normal University, Shanghai 200062, China; Institute of Magnetic Resonance and Molecular Imaging in Medicine, East China Normal University, Shanghai 200062, China; Department of Radiology, The Affiliated Wuxi People's Hospital of Nanjing Medical University, Wuxi 214023, China; Shanghai Key Laboratory of Magnetic Resonance, School of Physics and Electronic Science, East China Normal University, Shanghai 200062, China; Institute of Magnetic Resonance and Molecular Imaging in Medicine, East China Normal University, Shanghai 200062, China; Department of Radiology, The Affiliated Wuxi People's Hospital of Nanjing Medical University, Wuxi 214023, China; Department of Neurology, The Affiliated Wuxi People's Hospital of Nanjing Medical University, Wuxi 214023, China; Department of Radiology, The Affiliated Wuxi People's Hospital of Nanjing Medical University, Wuxi 214023, China

**Keywords:** Parkinson's disease, glymphatic system, cerebral iron deposition, choroid plexus volume, quantitative susceptibility mapping

## Abstract

Cerebral iron deposition is a pathological hallmark of Parkinson's disease. Notably, Parkinson's disease patients exhibit the characteristic neuroimaging features of enlarged choroid plexus volume (CPV) and diminished glymphatic function. While previous research has focused on potential iron influx dysregulation in Parkinson's disease, the critical question of whether impaired iron clearance stemming from choroid plexus and glymphatic system dysfunction constitutes a key mechanism underlying pathological iron accumulation remains unexplored. Therefore, the aim of this study was to investigate the relationship between cerebral iron deposition and glymphatic dysfunction in Parkinson's disease, and explored its clinical relevance. We hypothesized that impaired glymphatic clearance contributes to pathological iron accumulation in Parkinson's disease. This cross-sectional study enrolled 65 patients with mild-to-moderate Parkinson's disease and 38 age- and sex-matched healthy controls. Multimodal MRI was used to assess the CPV, diffusion tensor imaging analysis along the perivascular space (ALPS) index (indirectly reflecting glymphatic function) and quantitative susceptibility mapping-derived iron levels. The clinical evaluations included motor, cognitive and psychiatric assessments. Statistical analyses compared group differences, correlations, mediation effects and diagnostic performance via receiver operating characteristic analysis. Compared to healthy controls, Parkinson's disease patients exhibited bilateral CPV enlargement (*P* < 0.01), reduced ALPS indices (*P* < 0.05) and elevated iron deposition in the substantia nigra, red nucleus and putamen (*P* < 0.05). CPV negatively correlated with ALPS index (left hemisphere: *r* = −0.305, *P* = 0.014; right hemisphere: *r* = −0.357, *P* = 0.004). Notably, the degree of glymphatic dysfunction, manifested by either choroid plexus enlargement or a reduced ALPS index, was significantly correlated with regional iron deposition patterns, especially between the CPV and substantia nigra iron deposition (left hemisphere: *r* = 0.236, *P* = 0.029; right hemisphere: *r* = 0.233, *P* = 0.031). Mediation analysis revealed that ALPS and putamen iron deposition-mediated CPV affected daily living impairments and psychiatric symptoms. A comprehensive diagnostic model integrating neuroimaging, cognitive and psychiatric variables achieved near-perfect discrimination for mild-to-moderate Parkinson's disease (area under the receiver operating characteristic curve = 0.972, sensitivity = 100%, specificity = 97.4%). In conclusion, our results suggest that CPV enlargement and reduced ALPS index are closely linked to pathological iron deposition in Parkinson's disease. These biomarkers correlate with motor deficits, cognitive decline and psychiatric symptoms, highlighting their roles in disease progression. The integrated diagnostic model demonstrated exceptional accuracy, advocating multimodal approaches for Parkinson's disease management. These findings suggest that glymphatic modulation and iron chelation are potential therapeutic targets that warrant further longitudinal validation.

## Teaser

Chen *et al.* reported the possible new mechanism underlying cerebral iron deposition in Parkinson's disease that impairment in the glymphatic system, which removes waste and toxins from the brain, may contribute to the iron deposition, providing a new potential target for treating patients with Parkinson's disease.

## Graphical Abstract

**Graphical Abstract:**
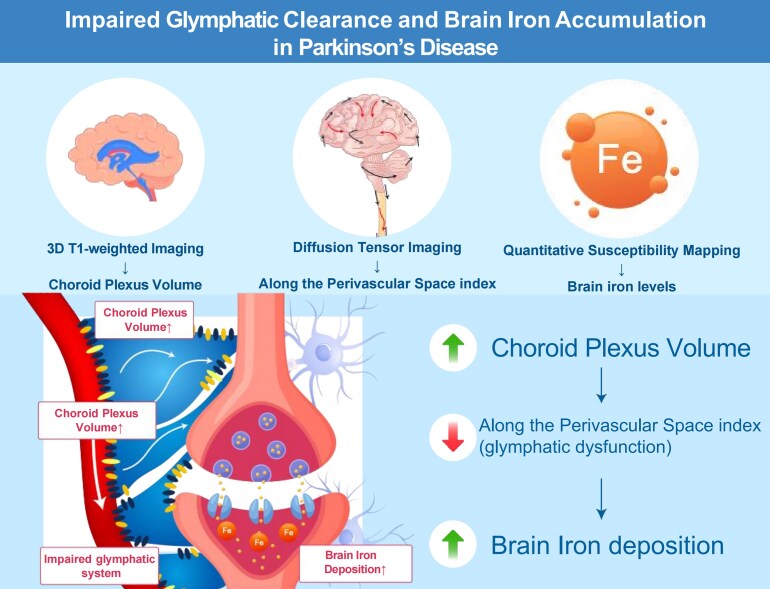

## Introduction

Parkinson's disease is the second most common neurodegenerative disease and is characterized by motor disorders including tremors, rigidity and bradykinesia.^1^ In the past decade, the global burden of Parkinson's disease has rapidly increased with an increasing number and proportion of elderly people.^2^ However, the mechanisms underlying the neurodegeneration in Parkinson's disease remain unclear.

In recent years, the pathogenic significance of cerebral iron deposition has been increasingly recognized as a central mechanistic contributor to neurodegeneration in Parkinson's disease.^3-5^ In A53T PD mouse models, iron dyshomeostasis in the substantia nigra (SN) exhibits chronological precedence over both dopaminergic neuronal silencing and motor phenotype emergence.^6^ Notably, iron chelation interventions during this prodromal phase delay neurodegeneration, suggesting that SN iron deposition may be an upstream regulator of α-synucleinopathy (α-syn) progression.^6^ Moreover, neuropathological progression in Parkinson's disease demonstrates a spatiotemporal expansion of cerebral iron deposition, exhibiting increased anatomical involvement and an amplified iron load in the affected brain regions.^7^ Although advanced quantitative MRI techniques, such as quantitative susceptibility mapping (QSM),^8,9^ have been used to detect cerebral iron deposition, the neurobiological drivers underlying progressive brain iron accumulation in Parkinson's disease remain unclear.

The progression of brain iron deposition is often associated with excessive input and clearance disorders.^10^ Accumulating evidence suggests that the pathological elevation of cerebral iron levels in Parkinson's disease may originate from dysregulated brain iron import mechanisms.^11^ Decreased serum iron coupled with elevated transferrin levels in Parkinson's disease may drive the pathological redistribution of peripheral iron into the brain, particularly into the SN, via systemic circulation.^10,12^ Parkinson's disease models exhibit divergent regulation of nigral iron transporters; increased expression of divalent metal transporter 1 coupled with decreased ferroportin 1 levels creates a unidirectional iron flux into the SN,^13^ establishing a redox imbalance through Fenton reaction-mediated oxidative damage.^14,15^ In addition, neuroimaging research has revealed a characteristic spatiotemporal trajectory of iron redistribution in Parkinson's disease, progressing from initial SN accumulation to basal ganglia involvement and culminating in cortical dissemination, which parallels clinical deterioration.^7^ Nevertheless, few studies have discussed whether defective cerebral iron clearance exists in Parkinson's disease, with current research predominantly focusing on iron influx dysregulation rather than impaired efflux pathways.

The cerebral iron homeostasis framework delineates the following regulatory systems: iron influx is predominantly governed by transferrin receptor-mediated transcytosis at the blood–brain barrier (BBB), whereas efflux is orchestrated through the glymphatic system mediated by the blood–cerebrospinal fluid barrier (BCSFB).^13,16-19^ The BCSFB, composed of choroid plexus epithelial cells, regulates cerebral iron homeostasis through passive paracellular clearance via interstitial fluid convection along the glymphatic routes.^13^ Therefore, the choroid plexus volume (CPV) indirectly reflects choroid plexus function, which is negatively correlated with choroid plexus permeability.^20^ The diffusion tensor image analysis along the perivascular space (DTI-ALPS) index reflects brain glymphatic function and may be a key indicator for measuring the brain iron clearance ability.^13,21^ According to neuroimaging evidence, patients with Parkinson's disease often exhibit increased CPV and a reduced ALPS index, both of which indicate severe glymphatic clearance dysfunction.^22-26^ However, few studies have explored the relationship between choroid plexus dysfunction, glymphatic dysfunction and brain iron deposition in Parkinson's disease.

We hypothesized that cerebral iron deposition in patients with Parkinson's disease is associated with impaired brain iron clearance mechanisms mediated by glymphatic dysfunction. In this study, we investigated the differences in the CPV, ALPS index and cerebral iron deposition detected using QSM between patients with Parkinson's disease and healthy control (HC). Subsequently, we examined the correlations between abnormally elevated iron deposition in key deep grey matter nuclei in patients with Parkinson's disease using both the CPV and ALPS indices. Overall, this study aimed to employ multimodal neuroimaging approaches to investigate the role of cerebral iron clearance dysfunction in driving pathological cerebral iron deposition progression in Parkinson's disease and the clinical correlates of these neuroimaging abnormalities.

## Materials and methods

### Subjects

A total of 70 patients with Parkinson's disease were enrolled consecutively through the Movement Disorders Clinic, Affiliated Wuxi People's Hospital of Nanjing Medical University (Wuxi, China), from August 2021 to August 2023. This study included an age- and sex-matched control group of 38 older adults. Further, this study strictly followed the ethical guidelines of the Declaration of Helsinki and was approved by the Ethics Committee of the Affiliated Wuxi People's Hospital of Nanjing Medical University. All participants provided written informed consent before the study began.

The inclusion criteria for patients with Parkinson's disease were as follows: (i) diagnosis confirmed per the clinical diagnostic criteria for Parkinson's disease^27^; (ii) age > 40 years at enrolment, (iii) right hand dominance and (iv) willingness to participate with signed consent documentation.

The exclusion criteria for patients with Parkinson's disease were as follows: (i) history of severe head trauma or any neurological/psychiatric disorders; (ii) inability to complete MRI examinations or clinical evaluations; (iii) abnormal findings detected on routine MRI; (iv) educational attainment below the primary school level and (v) poor imaging quality precluding postprocessing analysis.

### Clinical features

Following MRI, demographic and clinical data, including age, sex, educational attainment, disease duration, and the levodopa equivalent daily dose, were systematically recorded for all patients with Parkinson's disease. Two highly experienced neurologists specializing in Parkinson's disease administered the following assessments to the patients with Parkinson's disease: Unified Parkinson's Disease Rating Scale (UPDRS) Parts II and III, Hoehn & Yahr Staging (H&Y Scale), Mini-Mental State Examination (MMSE), Montreal Cognitive Assessment (MoCA), Freezing of Gait Questionnaire (FOG-Q), REM Sleep Behavior Disorder Screening Questionnaire (RBDSQ), Hamilton Anxiety Rating Scale (HAM-A) and Hamilton Depression Rating Scale (HAM-D). A similar battery of assessments was administered to HCs.

### MRI data

All participants underwent 3.0 T MRI (Siemens Prisma, Germany) in the morning using a 20-channel head coil. To minimize motion artefacts and acoustic noise, each subject was fitted with foam padding and MRI-compatible headphones prior to scanning. Patients with Parkinson's disease underwent brain MRI in the off-medication state following 12-h of withdrawal of dopaminergic therapy.

The parameters of the three-dimensional T1-weighted magnetization prepared rapid acquisition gradient echo (3D-T1 MP-RAGE) sequence, the DTI sequence and the QSM sequence were available in Supplementary Material 1.

### Choroid plexus volume calculation

FastSurfer (https://github.com/Deep-MI/FastSurfer) employed 3D-T1 MP-RAGE images for segmentation to obtain masks of the lateral ventricles and choroid plexus, with concurrent calculation of the total intracranial volume (TIV). Subsequently, a Gaussian mixture model was applied to refine the choroid plexus segmentation.^28^ Furthermore, all delineation results of choroid plexus segmentation were reviewed and manually corrected by two independent raters. Finally, choroid plexus volumetry was performed using ITK-SNAP (version 4.2.2; www.itksnap.org), with inter-rater discrepancies exceeding 15% resolved through a consensus review by a senior neuroradiologist. To mitigate the influence of interindividual differences in TIV on CPV measurements, CP was defined as the ratio of CPV/TIV, which represented the relative value of CPV for subsequent statistical analyses.

### ALPS analysis

#### DTI preprocessing

Diffusion data were processed using the FMRIB Software Library (FSL, version 6.0.1; www.fmrib.ox.ac.uk/fsl) with standard pipelines: (i) TOPUP for field inhomogeneity correction (b0 images, AP/PA encoding); (ii) EDDY for eddy current and motion correction; (iii) MP-PCA denoising and Gibbs unringing for artefact reduction; (iv) DTIFIT for tensor calculation [generating fractional anisotropy (FA), Dxx, Dyy, Dzz maps] and (v) non-linear registration to the Montreal Neurological Institute (MNI) space (JHU-ICBM-FA template via FLIRT/FNIRT) and spatial normalization.

#### Region of interest definition

Standardized 5-mm spherical regions of interest (ROIs) were placed at the ventricular body level in the normalized MNI space, targeting (i) projection fibres: Bilateral superior corona radiata (SCR); and (ii) association fibres: Bilateral superior longitudinal fasciculus (SLF). The template coordinates were as follows: Left SCR (116,110,99), right SCR (64,110,99), left SLF (128,110,99) and right SLF (51,110,99).

#### ALPS quantification

The ALPS index was computed as:


$$\;\mathrm{ALPS}\;\mathrm{index}=\frac{\mathrm{mean}(\mathrm{Dx}x_{\mathrm{proj}},\mathrm{Dx}x_{\mathrm{assoc}})}{\mathrm{mean}(\mathrm{Dy}y_{\mathrm{proj}},\mathrm{Dz}z_{\mathrm{assoc}})}$$

The terms are defined as follows: Dxx_proj, left–right diffusivity (projection fibers); Dxx_assoc, left–right diffusivity (association fibers); Dyy_proj, anterior–posterior diffusivity (projection fibers); Dzz_assoc, superior–inferior diffusivity (association fibers).

### Quantitative susceptibility mapping

#### QSM postprocessing

QSM postprocessing was performed to obtain QSM maps using the MEDI_GUI toolbox (https://pre.weill.cornell.edu/mri/pages/qsm.html) in MATLAB R2024b,^29^ as follows: (i) run mode: advanced mode was selected to access all parameterization options; (ii) brain mask generation: extracted the binary brain mask from the first echo magnitude image using the brain extraction tool (BET) from FSL; (iii) phase fitting: estimated the local frequency shift from multi-echo phase data via linear least-squares fitting across echoes; (iv) phase unwrapping: phase images were unwrapped via the region growth algorithm; (v) background field removal: performed using projection onto dipole fields and (vi) susceptibility inversion: conducted with morphology enabled dipole inversion (*λ* = 1000, edge = 0.9) incorporating the modified error reduction algorithm with iterative Tikhonov regularization and spherical mean value (Kernel radius = 5 mm) to QSM accuracy.

#### Susceptibility value extraction

Two neuroradiologists specializing in neuroimaging manually delineated the ROI on QSM maps using ITK-SNAP to extract mean susceptibility values from the following iron-rich brain regions implicated in Parkinson's disease pathophysiology: bilateral SN, red nucleus (RN), caudate nucleus (CN), putamen (PUT) and globus pallidus (GP).^30,31^ Inter-rater discrepancies exceeding 15% were resolved by a third senior neuroradiologist through a consensus review.

### Statistical analysis

All the statistical analyses were performed using SPSS (version 26.0, IBM Corp., Armonk, NY, USA). The significance threshold was set at *P* < 0.05, with the Bonferroni correction applied for multiple comparisons when applicable. Demographic and clinical characteristics are summarized as mean ± standard deviation for continuous variables.

Normality of the data distribution was assessed using the Shapiro–Wilk test. Group comparisons (Parkinson's disease versus HC) were conducted using the two-sample *t*-test or Mann–Whitney U-test (for continuous variables, based on normality) or the chi-square exact test (for categorical variables).

Adjusting for age, gender and educational attainment as covariates, partial correlation analyses were performed to assess relationships among neuroimaging biomarkers (CP, ALPS index and QSM values) within the Parkinson's disease group, HC group and combined cohort. Correlations between age and neuroimaging biomarkers of Parkinson's disease patients were assessed using the Pearson method. Then, we used the partial correlation analyses to evaluate correlations between neuroimaging biomarkers and clinical characteristics in Parkinson's disease cohort with age, gender and educational attainment as covariates. Simple mediation models were constructed using the PROCESS macro to explore whether glymphatic dysfunction (ALPS index) or iron deposition (QSM values) mediated the effects of CP on the clinical outcomes. Bootstrapping with 5000 iterations was used to estimate 95% confidence intervals for the indirect effects.

Finally, receiver operating characteristic (ROC) curve analysis was performed to evaluate the discriminatory power of the neuroimaging biomarkers and clinical variables with statistical differences for Parkinson's disease diagnosis, reporting the area under the curve (AUC), 95% confidence interval (CI), sensitivity and specificity (determined by maximizing the Youden Index). DeLong's test was used to compare the AUCs of the models. All visualizations were generated using GraphPad Prism (version 8.0.2).

## Results

### Population and participant characteristics

The final cohort comprised 65 patients with Parkinson's disease and 38 HCs after excluding five participants due to suboptimal MRI image quality. All enrolled patients with Parkinson's disease were at mild-to-moderate disease stages (H&Y stage ≤ 3), since advanced-stage patients (H&Y stage > 3) often cannot meet the rigorous requirements for multimodal MRI acquisition. The sex distribution, age and educational attainment of the Parkinson's disease cohort were comparable to the HCs (all *P* > 0.05). However, significant between-group differences were observed in the cognitive assessments, with patients with Parkinson's disease showing markedly lower MoCA and MMSE scores (*P* < 0.001 and *P* = 0.049, respectively). Additionally, the Parkinson's disease group exhibited significantly higher severity scores for both depression and anxiety symptoms compared to controls (both *P* < 0.001). Further details are presented in Table 1.

**Table 1:** Comparative analysis of the demographic and clinical characteristics between groups

	Parkinson's disease (*n* = 65)	HC (*n* = 38)	*P*-value
Sex (M/F)	39/26	19/19	0.323
Age (years)	64.29 ± 8.68	62.61 ± 7.99	0.330
Education (years)	9.75 ± 2.97	9.55 ± 3.96	0.787
Disease duration (years)	4.08 ± 3.05	NA	NA
H&Y	2.03 ± 0.73	NA	NA
LEDD (mg)	404.81 ± 228.91	NA	NA
UPDRS-II	9.66 ± 4.67	NA	NA
UPDRS-III	21.88 ± 10.45	NA	NA
FOG-Q	3.12 ± 5.15	NA	NA
RBDSQ	3.14 ± 3.72	NA	NA
MoCA	25.18 ± 2.94	28.18 ± 1.37	<0.001***
MMSE	28.48 ± 1.53	28.97 ± 1.00	0.049*
HAM-D	12.43 ± 5.84	3.05 ± 1.97	<0.001***
HAM-A	19.52 ± 4.85	2.61 ± 1.76	<0.001***

Abbreviations: HC, healthy controls; H&Y, Hoehn & Yahr scale; LEDD, levodopa equivalent daily dose; UPDRS, Unified Parkinson's Disease Rating Scale; FOG-Q, Freezing of Gait Questionnaire; RBDSQ, REM Sleep Behavior Disorder Screening Questionnaire; MoCA, Montreal Cognitive Assessment; MMSE, Mini-Mental State Examination; HAM-D, 17-item Hamilton Depression Rating Scale; HAM-A, Hamilton Anxiety Rating Scale; NA, not applicable.

Significance levels: *P  *<* 0.05, ***P  *<* 0.001 (Parkinson's disease versus HCs), Bonferroni-corrected.

### Neuroimaging biomarkers

#### Choroid plexus volume

**Figure 1:**
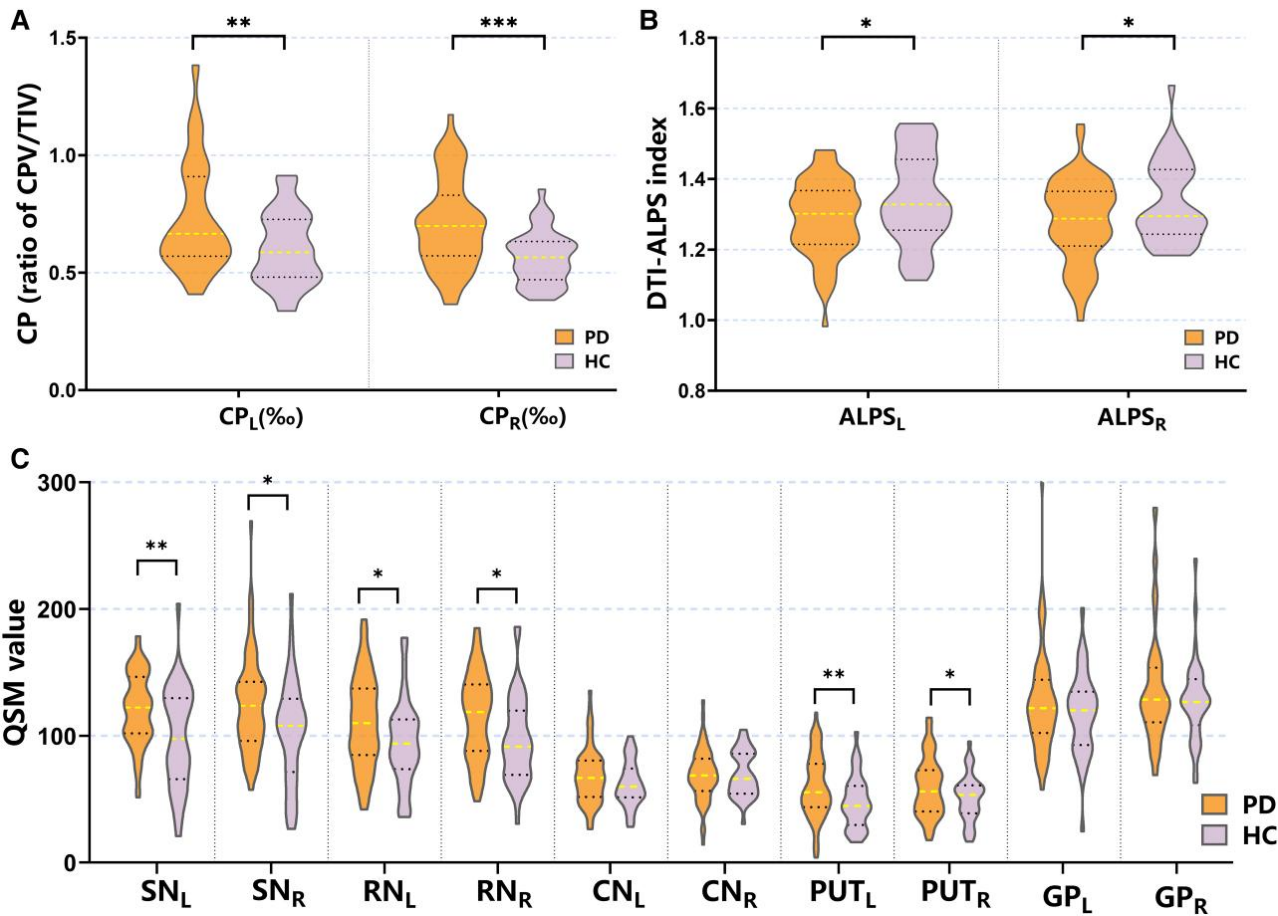
**Comparison of neuroimaging biomarkers between Parkinson's disease (*N* = 65) and HC (*N* = 38) with two-sample *t*-test.** (**A**) CP; (**B**) DTI-ALPS index; (**C**) susceptibility value in the SN, RN, CN, PUT and GP. Error bars represent the standard error of the mean. Asterisks indicate statistically significant group differences (*P < 0.05, **P < 0.01, ***P < 0.01). Abbreviations: CP, ratio of choroid plexus volume/total intracranial volume; L, left-hemispheric; R, right-hemispheric.

Quantification of the CPV revealed bilateral enlargement in patients with Parkinson's disease compared to that in HCs (left hemisphere: *P* = 0.003; right hemisphere: *P*  *<* 0.001, using Bonferroni correction). The TIV did not differ significantly between patients with Parkinson's disease and HCs (*P* = 0.246). To control for interindividual differences in TIV, CP was defined as the ratio of CPV/TIV. Comparative analysis revealed significantly elevated CP values in patients with Parkinson's disease relative to HCs, with bilateral significance (left hemisphere, *P* = 0.002; right hemisphere, *P*  *<* 0.001). The results are presented in Table 2 and Fig. 1A.

**Table 2:** Comparative analysis of neuroimaging biomarkers between groups

	Parkinson's disease (*n* = 65)	HC (*n* = 38)	*P*-value
Choroid plexus volume			
CPV_L_ (mm^3^)	1099.97 ± 411.96	871.79 ± 251.82	0.003**
CPV_R_ (mm^3^)	1054.34 ± 333.03	816.42 ± 182.96	<0.001***
TIV (mm^3^)	1 484 074.75 ± 165 924.37	1 446 202.89 ± 146 299.30	0.246
CP_L_ (‰)	0.73 ± 0.23	0.60 ± 0.15	0.002**
CP_R_ (‰)	0.71 ± 0.19	0.56 ± 0.11	<0.001***
DTI-ALPS index			
ALPS_L_	1.29 ± 0.11	1.34 ± 0.13	0.025*
ALPS_R_	1.28 ± 0.12	1.33 ± 0.11	0.024*
QSM			
SN_L_	121.01 ± 28.09	99.32 ± 39.72	0.002**
SN_R_	124.98 ± 37.95	103.89 ± 41.39	0.012*
RN_L_	111.23 ± 35.78	94.46 ± 33.74	0.021*
RN_R_	115.37 ± 33.63	97.40 ± 34.06	0.011*
CN_L_	68.13 ± 21.83	62.04 ± 17.84	0.148
CN_R_	69.04 ± 20.22	69.57 ± 17.95	0.895
PUT_L_	59.78 ± 24.69	47.66 ± 20.51	0.009**
PUT_R_	59.61 ± 23.05	50.98 ± 18.42	0.039*
GP_L_	126.34 ± 39.99	117.87 ± 32.91	0.272
GP_R_	136.49 ± 44.13	127.71 ± 32.59	0.289

Abbreviations: HC, healthy controls; CPV, choroid plexus volume; TIV, total intracranial volume; CP, ratio of CPV/TIV; DTI-ALPS, diffusion tensor image analysis along the perivascular space; QSM, quantitative susceptibility mapping; SN, substantia nigra; RN, red nucleus; CN, caudate nucleus; PUT, putamen; GP, globus pallidus; L, left-hemispheric; R, right-hemispheric.

Significance levels: *P  *<* 0.05, **P  *<* 0.01, ***P  *<* 0.001 (Parkinson's disease versus HC), Bonferroni-corrected.

#### DTI-ALPS index

Patients with Parkinson's disease exhibited significantly reduced ALPS indices in both hemispheres compared to HCs (left hemisphere, *P* = 0.025; right hemisphere, *P* = 0.024, using Bonferroni correction). See Table 2 and Fig. 1B for details.

#### Quantitative susceptibility mapping

QSM revealed significantly higher susceptibility values in the SN (left hemisphere: *P* = 0.002; right hemisphere: *P* = 0.012, using Bonferroni correction), RN (left hemisphere: *P* = 0.021; right hemisphere: *P* = 0.011) and PUT (left hemisphere: *P* = 0.009; right hemisphere: *P* = 0.039) bilaterally in patients with Parkinson's disease compared with controls. No between-group differences were observed in susceptibility values for CN or GP (both *P* > 0.05). Quantitative comparisons of the regional susceptibility values are provided in Table 2 and Fig. 1C.

### Cross-modal relationships between neuroimaging biomarkers

#### Correlations between CP and the ALPS index

**Figure 2:**
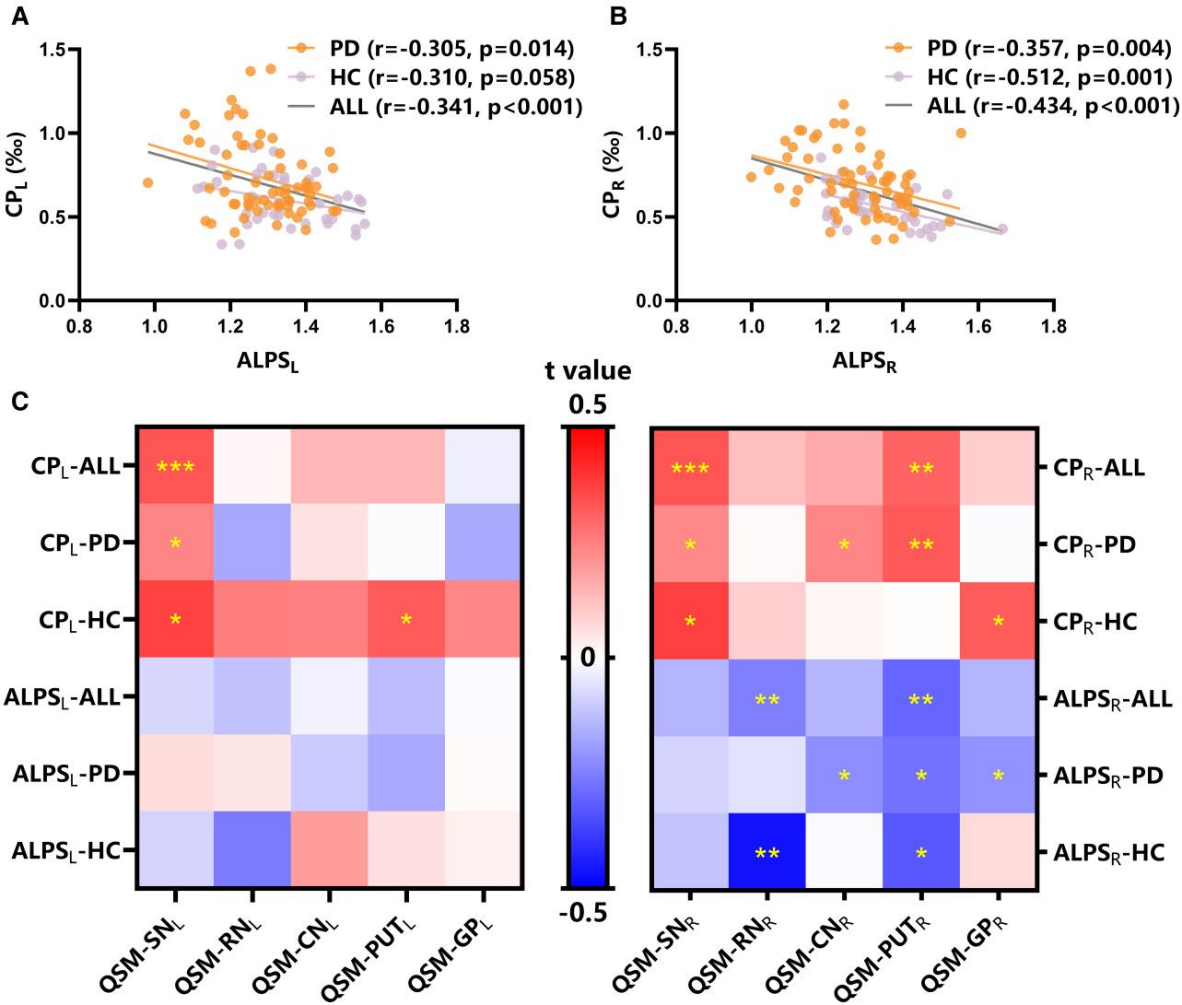
**Cross-modal correlations between neuroimaging biomarkers.** (**A, B**) Partial correlation analysis between CP and the DTI-ALPS index in Parkinson's disease cohort (*N* = 65); (**C**) partial correlation analysis between glymphatic biomarkers and regional susceptibility values in Parkinson's disease cohort (*N* = 65). QSM represents the QSM values of the corresponding brain regions. Asterisks indicate statistically significant group differences (*P < 0.05, **P < 0.01, ***P < 0.01). Abbreviations: All, all participants; CP, ratio of choroid plexus volume/total intracranial volume; L, left-hemispheric; R, right-hemispheric.

At the left hemispheric level, the CP exhibited significant negative correlations with the ALPS index in both the Parkinson's disease group (*r* = −0.305, *P* = 0.014) and the combined cohort (*r* = −0.341, *P*  *<* 0.001), whereas only a non-significant trend was observed in the HC group (*r* = −0.310, *P* = 0.058). The results are presented in Supplementary Table 1 and Fig. 2A.

At the right hemispheric level, the CP demonstrated significant negative correlations with the ALPS index across all cohorts: the Parkinson's disease group (*r* = −0.357, *P* = 0.004), the HC group (*r* = −0.512, *P* = 0.001) and the entire sample (*r* = −0.434, *P*  *<* 0.001). The results are presented in Supplementary Table 1 and Fig. 2B.

#### Correlations between glymphatic markers and regional iron accumulation

Our analyses revealed distinct patterns of glymphatic susceptibility relationships across the groups. We observed consistent positive associations between SN susceptibility and CP across all study groups, with bilateral significance (Parkinson's disease group: left hemisphere: *r* = 0.236, *P* = 0.029; right hemisphere: *r* = 0.233, *P* = 0.031; HC group: left hemisphere: *r* = 0.367, *P* = 0.012; right hemisphere: *r* = 0.373, *P* = 0.011; combined cohort: left hemisphere: *r* = 0.333, *P*  *<* 0.001; right hemisphere: *r* = 0.332, *P*  *<* 0.001). Additionally, in the HC group, left PUT susceptibility showed a significant positive correlation with ipsilateral CP (*r* = 0.32, *P* = 0.025), whereas right RN susceptibility was inversely associated with the right ALPS index in both the HC group (*r* = −0.466, *P* = 0.002) and the combined cohort (*r* = −0.25, *P* = 0.005). Patients with Parkinson's disease exhibited more complex right-hemisphere glymphatic susceptibility associations; CN susceptibility demonstrated a positive correlation with CP (*r* = 0.25, *P* = 0.029) and a negative correlation with the ALPS index (*r* = −0.22, *P* = 0.038). Right PUT susceptibility displayed consistent positive correlations with the right CP in patients with Parkinson's disease (*r* = 0.325, *P* = 0.004) and all participants (*r* = 0.304, *P* = 0.001), and showed uniformly negative associations with the right ALPS index across the Parkinson's disease group (*r* = −0.278, *P* = 0.012), HC group (*r* = −0.329, *P* = 0.022) and the full sample (*r* = −0.301, *P* = 0.001). Finally, right GP susceptibility was negatively correlated with the right ALPS index in the patients with Parkinson's disease (*r* = −0.212, *P* = 0.045), whereas it showed a positive correlation with the right CP in the HC group (*r* = 0.319, *P* = 0.025). No significant correlations were observed between other glymphatic markers and regional susceptibility values (*P* > 0.05). The details are presented in Supplementary Table 1 and Fig. 2C.

### Correlation between neuroimaging biomarkers and clinical characteristics related to Parkinson's disease

**Figure 3:**
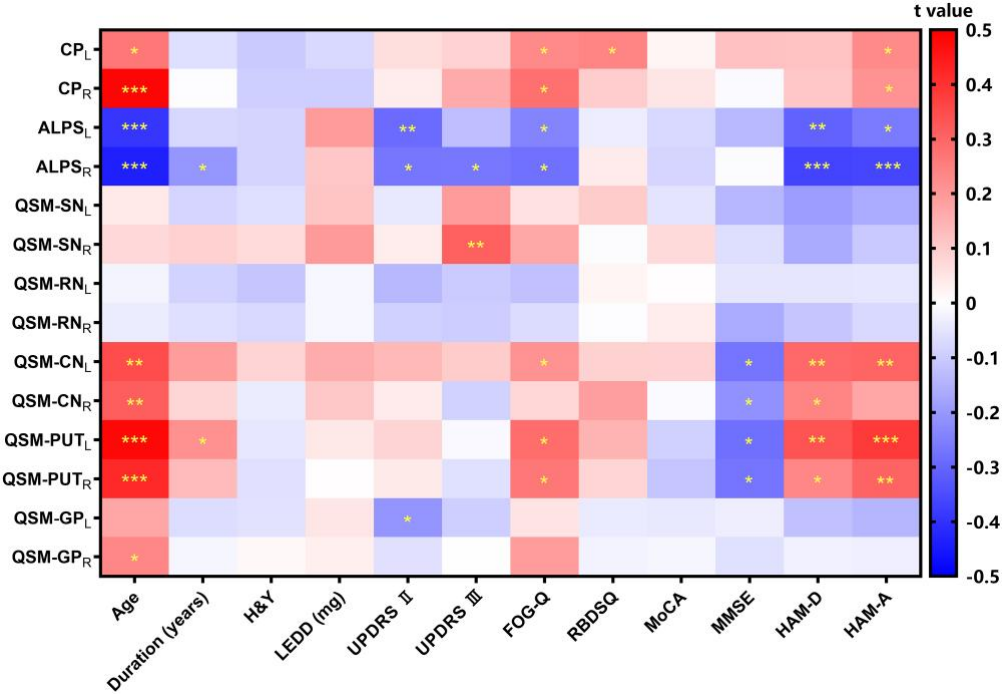
**Partial correlation analysis between neuroimaging biomarkers and clinical characteristics related to Parkinson's disease (*N* = 65).** QSM represents the QSM values of the corresponding brain regions. Asterisks indicate statistically significant group differences (*P < 0.05, **P < 0.01, ***P < 0.01). Abbreviations: CP, ratio of choroid plexus volume/total intracranial volume; LEDD, levodopa equivalent daily dose; L, left-hemispheric; R, right-hemispheric.

In Parkinson's disease patients, age was negatively correlated with bilateral ALPS indices (left hemisphere: *r* = −0.396, *P* < 0.001; right hemisphere: *r* = −0.438, *P* < 0.001), while showing positive associations with CP (left hemisphere: *r* = 0.264, *P* = 0.017; right hemisphere: *r* = 0.487, *P* < 0.001), CN susceptibility (left hemisphere: *r* = 0.348, *P* = 0.002; right hemisphere: *r* = 0.314, *P* = 0.005), PUT susceptibility (left hemisphere: *r* = 0.483, *P* < 0.001; right hemisphere: *r* = 0.419, *P* < 0.001) and right GP susceptibility (*r* = 0.235, *P* = 0.03). Disease duration was inversely associated with the right ALPS index (*r* = −0.207, *P* = 0.049) but positively correlated with left putaminal susceptibility (*r* = 0.215, *P* = 0.042). UPDRS-II scores were negatively correlated with bilateral ALPS indices (left hemisphere: *r* = −0.292, *P* = 0.009; right hemisphere: *r* = −0.269, *P* = 0.015) and left GP susceptibility (*r* = −0.208, *P* = 0.048), whereas UPDRS-III scores were associated with a reduced right ALPS index (*r* = −0.265, *P* = 0.016) and elevated right SN susceptibility (*r* = 0.309, *P* = 0.006). FOG assessments revealed positive associations with bilateral CP (left hemisphere: *r* = 0.229, *P* = 0.033; right hemisphere: *r* = 0.277, *P* = 0.013), PUT susceptibility (left hemisphere: *r* = 0.285, *P* = 0.011; right hemisphere: *r* = 0.263, *P* = 0.017) and left CN susceptibility (*r* = 0.213, *P* = 0.044), alongside negative correlations with bilateral ALPS indices (left hemisphere: *r* = −0.243, *P* = 0.025; right hemisphere: *r* = −0.280, *P* = 0.012). RBD severity was positively correlated with left CP (*r* = 0.238, *P* = 0.028). The MMSE scores were inversely related to bilateral CN (left hemisphere: *r* = −0.272, *P* = 0.014; right hemisphere: *r* = −0.216, *P* = 0.042) and PUT susceptibility (left hemisphere: *r* = −0.281, *P* = 0.012; right hemisphere: *r* = −0.271, *P* = 0.015). Depression severity was positively correlated with bilateral CN (left hemisphere: *r* = 0.293, *P* = 0.009; right hemisphere: *r* = 0.241, *P* = 0.027) and putaminal susceptibility (left hemisphere: *r* = 0.334, *P* = 0.003; right hemisphere: *r* = 0.234, *P* = 0.03) but negatively correlated with ALPS indices (left hemisphere: *r* = −0.308, *P* = 0.006; right hemisphere: *r* = −0.367, *P* < 0.001). Finally, anxiety scores were positively associated with bilateral CP (left: *r* = 0.228, *P* = 0.034; right: *r* = 0.212, *P* = 0.045), PUT susceptibility (left hemisphere: *r* = 0.386, *P* < 0.001; right hemisphere: *r* = 0.303, *P* = 0.007) and left CN susceptibility (*r* = 0.301, *P* = 0.007), and negatively correlated with bilateral ALPS indices (left hemisphere: *r* = −0.259, *P* = 0.006; right hemisphere: *r* = −0.363, *P* < 0.001). All other neuroimaging-clinical correlations were below the predefined significance threshold (*P* > 0.05). Detailed information is presented in Supplementary Table 2 and Fig. 3.

### Mediation analysis

#### CP influenced UPDRS-II scores through modulation of the ALPS index in patients with Parkinson's disease

**Figure 4:**
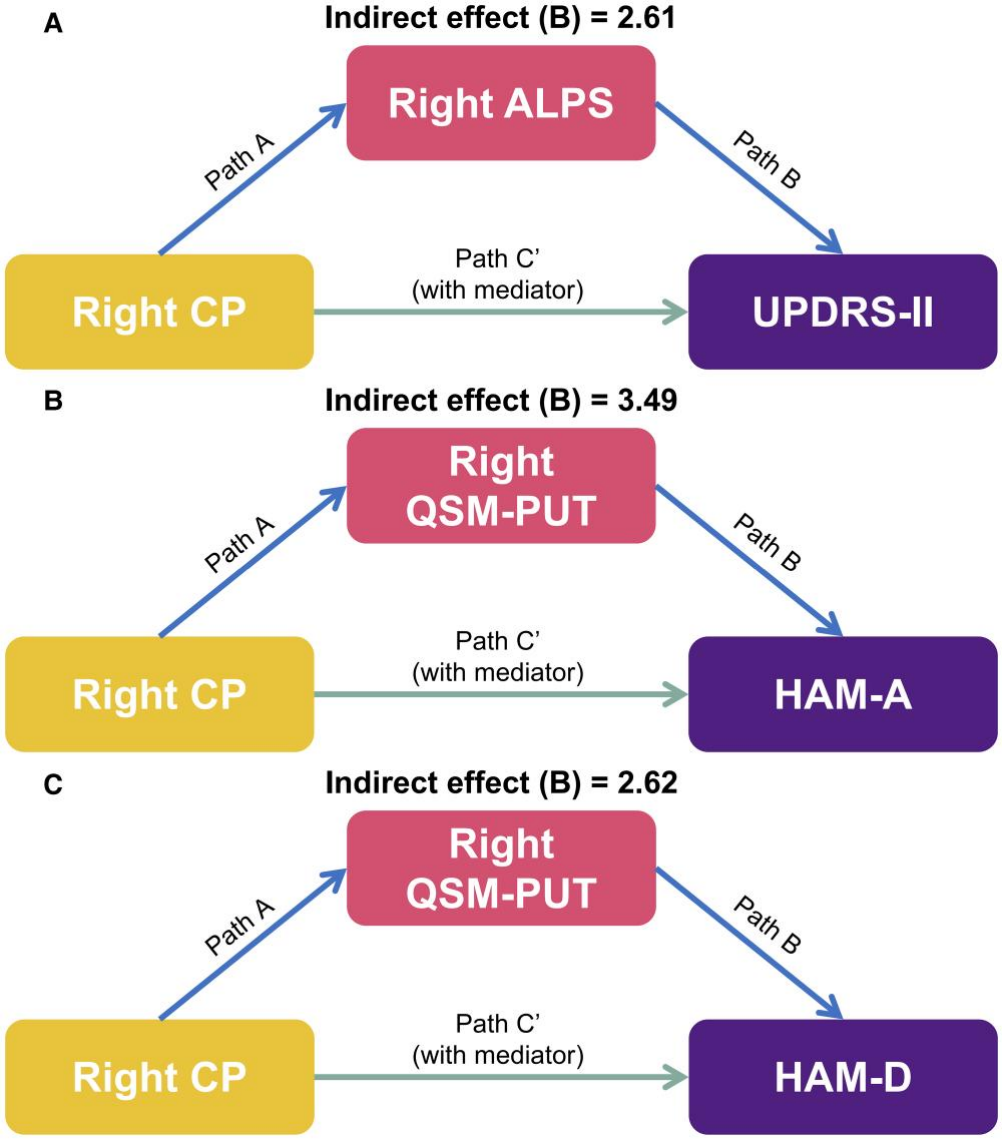
**Mediation analysis using simple mediation models in patients with Parkinson's disease (*N* = 65).** (**A**) The mediation effects of the right ALPS index between the right CPV and UPDRS-II score; (**B**) the mediation effects of right PUT iron deposition between the right CPV and HAM-A score; (**C**) the mediation effects of right PUT iron deposition between the right CPV and HAM-D score. Abbreviations: QSM-PUT, susceptibility value of the putamen.

A significant indirect effect of the right CP on UPDRS-II scores (used to assess impairments in activities of daily living) via the right ALPS index was observed (effect = 2.61, SE = 1.80, 95% CI [0.23, 7.12]). Right CP negatively predicted right ALPS (*β* = −0.22, *P* = 0.004), which in turn was associated with lower UPDRS-II scores (*β* = −11.81, *P* = 0.028). The absence of significant direct or total effects (*P* > 0.59) supported full mediation, as illustrated in Fig. 4A.

#### CP contributed to neuropsychiatric symptoms via modulation of PUT iron deposition in Parkinson's disease

Mediation analysis demonstrated a significant indirect effect of the right CP on anxiety and depression symptoms through iron deposition in the right PUT in patients with Parkinson's disease. Right CP positively predicted right PUT susceptibility (*β* = 39.85, *P* = 0.008), which in turn was associated with higher HAM-A scores (*β* = 0.09, *P* = 0.047), with a significant indirect effect (effect = 3.49, SE = 2.12, 95% CI [0.30, 8.51]), as illustrated in Fig. 4B.

Similarly, right PUT susceptibility mediated the relationship between right CP and HAM-D scores (*β* = 0.07, *P* = 0.046), showing a significant indirect effect (effect = 2.62, SE = 1.59, 95% CI [0.19, 6.44]). The absence of significant direct or total effects (both *P* > 0.39) in both models supported full mediation, as illustrated in Fig. 4C.

### Neuroimaging biomarkers, cognitive scores and psychiatric symptoms in the combined diagnostic efficacy for Parkinson's disease

**Figure 5:**
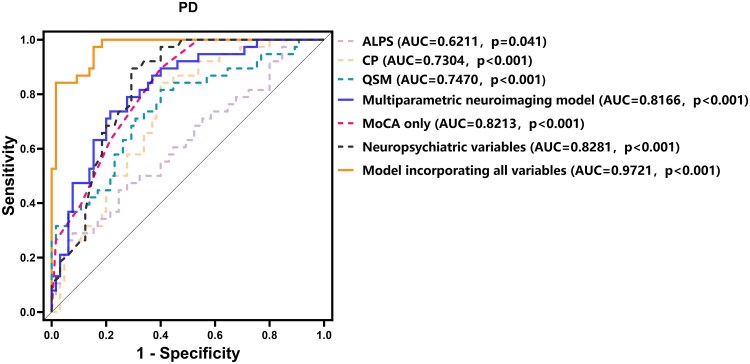
**Diagnostic performance of multiple models for mild-to-moderate-stage Parkinson's disease using ROC curve analysis (*N* = 65).** The diagnostic model for Parkinson's disease incorporating all variables significantly outperformed all univariate models, achieving near-perfect discriminative capability (AUC = 0.972). The multiparametric neuroimaging model integrated ALPS + CP + QSM. The neuropsychiatric variables model integrated HAM-D + HAM-A. Abbreviations: CP, ratio of choroid plexus volume/total intracranial volume.

ROC curve analysis was used to evaluate the diagnostic performance of multiple models for mild-to-moderate stage Parkinson's disease. The ALPS model demonstrated modest performance (AUC = 0.621, 95% CI: 0.508–0.735), a sensitivity of 66.2% and a specificity of 50.0%. The CP model showed improved discrimination (AUC = 0.730, 95% CI: 0.634–0.826), with 60.0% sensitivity and 73.7% specificity. Further enhancement was observed with the QSM model (AUC = 0.747, 95% CI: 0.647–0.847), with 73.8% sensitivity with 55.3% specificity. Neuroimaging-based models comprising neuroimaging biomarkers exhibited strong diagnostic utility. The multiparametric neuroimaging model achieved an AUC of 0.817 (95% CI: 0.734–0.899) with 80.0% sensitivity and 65.8% specificity. The MoCA score (AUC = 0.821; 95% CI, 0.743–0.899) had high specificity (89.5%), but limited sensitivity (40.0%), while the neuropsychiatric variable model incorporating the HAM-D and HAM-A scores (AUC = 0.828, 95% CI: 0.751–0.905) prioritized sensitivity (81.5%) over specificity (42.1%). Notably, the comprehensive model incorporating all variables outperformed all other models with near-perfect discrimination (AUC = 0.972, 95% CI: 0.947–0.997). It achieved 100% sensitivity and 97.4% specificity, demonstrating a robust ability to identify true cases while minimizing the number of false positives. DeLong's test revealed that the comprehensive model surpassed all other models (*P* < 0.001 versus ALPS, CP, QSM, multiparametric neuroimaging model, MoCA-only and neuropsychiatric variables). The results are shown in Fig. 5.

## Discussion

This study applied multimodal neuroimaging techniques to systematically elucidate the relationship between cerebral iron deposition and glymphatic dysfunction in Parkinson's disease, revealing significant associations between an enlarged CPV, reduced ALPS index and pathological iron accumulation. Furthermore, these neuroimaging biomarkers demonstrated robust correlations with clinical symptomatology, and edition analyses demonstrated that glymphatic dysfunction and iron deposition mediate the relationship between CPV and clinical symptoms. Finally, the integrated diagnostic model, which incorporated comprehensive cognitive assessments, psychiatric symptom evaluations and neuroimaging biomarkers, exhibited remarkable diagnostic efficacy for mild-to-moderate-stage Parkinson's disease. These findings provide new insights into the mechanisms underlying iron dyshomeostasis in Parkinson's disease and highlight potential therapeutic targets.

### Choroid plexus enlargement may contribute to glymphatic dysfunction in Parkinson's disease

Our observation of bilateral CPV enlargement in patients with Parkinson's disease is consistent with previous neuroimaging studies that reported choroid plexus hypertrophy in neurodegenerative diseases, including Alzheimer's disease and PD,^20,22^ which usually supports that the increase of choroid plexus volume usually represents compensatory tissue hypertrophy or oedema, thus reflecting the decline of choroid plexus function. Recently, it has been proposed that choroid plexus enlargement in Alzheimer's disease may reflect compensatory mechanisms due to impaired cerebrospinal fluid (CSF) clearance,^20^ a hypothesis that may extend to Parkinson's disease. Some review articles conceptualized CPV as a key regulator in maintaining central nervous system homeostasis, establishing it as a crucial biomarker for evaluating glymphatic system functionality.^32,33^ Notably, our study directly linked CPV enlargement to glymphatic dysfunction (reduced ALPS index) in Parkinson's disease, which has been consistently reported in both Alzheimer's disease and multiple sclerosis.^34,35^ This suggests that choroid plexus pathology may disrupt interstitial fluid convection along the perivascular spaces, a critical pathway for brain iron clearance.^13^ This finding complements recent studies, which reported glymphatic impairment in Parkinson's disease using DTI-ALPS analysis but did not explore its relationship with CPV, and uniquely established a direct negative correlation between CPV and the ALPS index, suggesting that choroid plexus enlargement may exacerbate glymphatic dysfunction by altering CSF turnover or interstitial fluid dynamics.^24,25^

### Iron deposition in Parkinson's disease may be associated with impaired glymphatic clearance function

The elevated iron deposition in the SN, RN and PUT of patients with Parkinson's disease is consistent with prior QSM studies.^7,30,31^ However, the absence of significant iron accumulation in the GP contrasted with some reports,^31^ possibly attributable to the exclusion of advanced-stage Parkinson's disease patients (H-Y stage > 3) in our cohort. Additionally, our study is the first to demonstrate that iron accumulation in parts of these regions correlates positively with CPV and negatively with the ALPS index. This aligns with the theory that brain iron homeostasis depends on a balanced influx (via transferrin receptors) and efflux (via glymphatic clearance). While previous studies have attributed Parkinson's disease iron dysregulation primarily to upregulated BBB iron influx,^11^ our findings provide the first systematic evidence implicating glymphatic-mediated iron clearance failure as a driver of pathological iron deposition.

Furthermore, only iron deposition in the SN demonstrated a positive correlation with bilateral CPV, and the susceptibility of other regions exhibited right hemisphere predominance in association with impaired glymphatic clearance function, which may reflect the characteristic clinical and pathological asymmetries observed in Parkinson's disease,^36^ but unfortunately, this study did not record the lateralization information of Parkinson's disease patients in detail. In addition, compared with Parkinson's disease group, a higher correlation was found between CPV and SN iron deposition in HC group, which may be because of the imbalance between the input and output of SN iron and the greater compensatory effect of choroid plexus in Parkinson's disease. A recent study employed both the ALPS and QSM multimodal neuroimaging approaches to investigate Parkinson's disease.^37^ However, their analysis was restricted to iron deposition in the SN alone and overlooked the potential relationship between the glymphatic system and SN iron accumulation; correlation analysis between these measures was not performed. Our findings demonstrate that SN iron deposition in patients with Parkinson's disease was more closely associated with choroid plexus function than with the ALPS index. Finally, the animal study had demonstrated that glymphatic dysfunction contributes to α-syn accumulation in Parkinson's disease,^38^ which was consistent with our findings because of previous reports of frequent iron-α-syn co-deposition in the SN of Parkinson's disease.^11^

### The association between neuroimaging biomarkers and clinical manifestations in Parkinson's disease

Age exhibited robust correlations with multiple neuroimaging measures, including negative associations with the ALPS index and positive correlations with CPV and iron deposition in the CN and PUT groups. These observations are consistent with previous studies demonstrating an age-related decline in glymphatic function and choroid plexus hypertrophy.^39-41^ Notably, our data extend these findings by showing that age-associated glymphatic dysfunction may exacerbate iron accumulation in regions vulnerable to Parkinson's disease, suggesting a synergistic effect of aging and Parkinson's disease pathology on brain iron homeostasis. This supports the ‘two-hit’ hypothesis, where aging (hit one) primes the brain for Parkinson's disease-specific iron dysregulation (hit two).^10,30^

Longer disease duration was correlated with higher PUT iron deposition and a reduced right ALPS index, reinforcing the role of glymphatic impairment in progressive iron accumulation. This parallels the neuropathological evidence of spatiotemporal iron expansion in Parkinson's disease.^7^ However, our study uniquely links this progression to declining glymphatic efficiency. The lack of a significant association between disease duration and CPV implies that choroid plexus hypertrophy may occur early in Parkinson's disease, preceding measurable glymphatic function decline, a hypothesis that warrants longitudinal validation.

The UPDRS-III scores correlated with right SN iron deposition and the right ALPS index, consistent with the role of the SN in motor dysfunction reported in previous studies.^42^ Mediation analysis further revealed that the right ALPS index fully mediated the effect of CPV on UPDRS-II scores, underscoring glymphatic dysfunction as a potential key driver of impaired QoL in patients with Parkinson's disease, a finding aligned with established research evidence.^43^

The severity of FOG was associated with bilateral glymphatic dysfunction and PUT iron deposition, highlighting the pivotal role of PUT in the pathogenesis of FOG in Parkinson's disease. Previous studies have suggested that FOG may be associated with aberrantly enhanced functional connectivity between the PUT and amygdala, suggesting that this pathological mechanism may be mediated through iron deposition-induced functional disruption of the PUT.^44^ However, in contrast to our findings, some studies have reported no significant differences in the ALPS index between patients with Parkinson's disease with and without FOG.^45^ This may be because Parkinson's disease with FOG additionally involves abnormalities in basal ganglia-cortical circuits and degeneration of the noradrenergic system, thereby attenuating the observed association with glymphatic dysfunction.^46,47^

Higher CN and PUT iron deposition were found to correlate with lower MMSE scores and higher HAM-D/HAM-A scores, supporting the role of striatal iron, not just the cortex, in cognitive impairment and psychiatric symptoms.^23,48-50^ Similarly, several studies have reported a negative relationship between striatal iron deposition and cognitive scores in patients with Parkinson's disease.^51,52^ Regarding mental disorders, the previous research identified a correlation between anxiety scores and reduced functional connectivity between the PUT and amygdala in Parkinson's disease.^53^ In contrast, previous PET studies demonstrated that reduced striatal dopamine transporter density predicts more severe psychiatric symptoms in patients with Parkinson's disease,^54^ a finding replicated in animal models.^55^ This study provides further evidence regarding the potential mechanisms related to PUT ferroptosis leading to mental disorders in Parkinson's disease, which were completely regulated by choroid plexus function according to mediation analysis.

### Diagnostic superiority of integrating multimodal neuroimaging biomarkers with clinical variables

The comprehensive model combining the CPV, ALPS index, QSM-derived iron deposition and neuropsychiatric assessments achieved near-perfect discrimination between mild-to-moderate-stage Parkinson's disease and HCs (AUC = 0.972), surpassing individual neuroimaging biomarker models such as the QSM (AUC = 0.747) or ALPS index alone (AUC = 0.621). This aligns with emerging evidence advocating for multimodal approaches in neurodegenerative disease diagnostics,^26,37^ as single biomarkers often fail to capture the multifactorial nature of Parkinson's disease pathology.^22,24^ Previous studies have also employed a combination of neuroimaging biomarkers (bilateral perivascular spaces and the ALPS index or the QSM of the bilateral SN and ALPS index) for ROC analysis in Parkinson's disease, yet achieved only moderate diagnostic accuracy (AUC = 0.85 and 0.801), likely due to the exclusion of crucial clinical parameters.^25,37^ The exceptional sensitivity (100%) and specificity (97.4%) of this model contrasted with the trade-offs observed in isolated assessments—the high specificity (89.5%) but limited sensitivity (40%) of the MoCA and inverse profile of neuropsychiatric variables—emphasizing the clinical value of integrated evaluation. Notably, the synergistic performance of CPV, the ALPS index and QSM highlighted their complementary roles: CPV enlargement reflected choroid plexus dysfunction, whereas ALPS index reduction directly quantified glymphatic impairment, providing a holistic proxy for iron clearance failure. While prior studies independently linked CPV to α-synucleinopathy or the ALPS index to Parkinson's disease severity,^22,24^ this study pioneered their combined use, demonstrating unprecedented diagnostic precision. These results underscore the diagnostic advantage of integrating multimodal data, and the exceptional performance of the combined model highlights its potential for clinical applications in mild-to-moderate-stage Parkinson's disease detection. Future validation in prodromal cohorts could further refine the utility of this model for early intervention, thereby addressing the critical unmet need in Parkinson's disease management.

### Limitations

This study has some limitations, as follows. (i) Sample size and generalizability: Since this was a single-centre exploratory investigation with a limited sample size, there are constraints on generalizability. The preliminary nature of these findings necessitates validation through multicentre studies with larger cohorts to establish broader clinical applicability. The exclusion of advanced-stage patients with Parkinson's disease further restricted applicability to mild-to-moderate cases, and the diagnostic model requires external validation in independent cohorts for clinical translation. In addition, the lack of lateralization information made it difficult to explain the rightization of the results in this paper. (ii) Cross-sectional design: the observational nature of the study precludes causal inferences between glymphatic dysfunction, iron deposition and clinical symptoms. Longitudinal studies are needed to validate temporal relationships, such as tracking prodromal Parkinson's disease cohorts (e.g. idiopathic RBD) to test directional relationships among baseline CPV, follow-up ALPS decline and follow-up iron accumulation,^56^ or applying Parkinson's disease animal models with damaged glymphatic system to explore its effect on brain iron deposition. (iii) Differential analysis: glymphatic dysfunction and brain iron deposition are common in nearly all neurodegenerative diseases, but the relationship between glymphoid dysfunction and brain iron deposition in other neurodegenerative diseases was not discussed in this paper to determine whether it is a unique mechanism in Parkinson's disease. In the future, similar research can be considered in other neurodegenerative diseases such as Alzheimer's disease or multiple system atrophy. (iv) Potential measurement bias: manual delineation of QSM ROIs by neuroradiologists, despite consensus resolution, may have introduced subjectivity. Additionally, the ALPS index indirectly assessed glymphatic function, lacking direct measurement of CSF dynamics. Although previous studies have supported the robustness of ALPS by studying the high correlation between traditional glymphatic imaging and ALPS index,^57^ the recent study suggest that age-related white matter degeneration may increase the false positive rate of ALPS by 30% due to the ROI dependence of methodology,^58^ which was an unavoidable limitation even if age had been used as a covariate to assess the correlation. In future research, more robust methods to evaluate glymphatic system, such as traditional enhanced glymphatic MRI, could be applied to solve this problem. (v) Technical limitations: Although motion artefacts were minimized, subtle movements in patients with Parkinson's disease during MRI scanning may have affected data accuracy.

## Conclusion

This study demonstrated that impaired glymphatic clearance, as evidenced by choroid plexus enlargement and a reduced ALPS index, was closely associated with pathological iron deposition in key brain regions of patients with Parkinson's disease, suggesting a mechanistic link between glymphatic dysfunction and iron dyshomeostasis. The identified correlations between neuroimaging biomarkers (CPV, ALPS index and QSM-derived iron levels) and clinical manifestations, including motor deficits, cognitive decline and psychiatric symptoms, highlight their potential roles in tracking Parkinson's disease progression. Notably, the integrative diagnostic model, combining multimodal neuroimaging, cognitive assessments and psychiatric evaluations, achieved exceptional accuracy (AUC = 0.972), underscoring the clinical value of holistic approaches for Parkinson's disease diagnosis. These findings not only advance our understanding of iron clearance failure in Parkinson's disease pathogenesis but also suggest therapeutic targets, such as glymphatic modulation and iron chelation, to mitigate neurodegeneration. Future longitudinal and mechanistic studies are warranted to validate these associations and to provide insights into clinical interventions.

## Supplementary Material

fcaf365_Supplementary_Data

## Data Availability

The corresponding authors will provide the raw data upon request without reservation. All code applied for data analysis in this manuscript could be found in Supplementary Material 2.
