# Supplementary material for: Glymphatic dysfunction as a potential driver of cerebral iron deposition in Parkinson's disease
Source: Brain Commun. 2025 Sep 23;7(5):fcaf365. doi: 10.1093/braincomms/fcaf365 (PMC12507083; doi:10.1093/braincomms/fcaf365)
Supplement: fcaf365_Supplementary_Data [file fcaf365_supplementary_data.docx]

| **Supplementary Table 1** **Correlation analysis between neuroimaging biomarkers** | | | | | | |
| --- | --- | --- | --- | --- | --- | --- |
|  | **ALPS** | **QSM-SN** | **QSM-RN** | **QSM-CN** | **QSM-PUT** | **QSM-GP** |
| **Left-hemisphere** | | | | | | |
| CP | -0.314 (0.001^***^) | 0.333 (0.001^***^) | 0.015 (0.440) | 0.138 (0.082) | 0.141 (0.077) | -0.034 (0.367) |
| CP_PD_ | -0.305 (0.014^*^) | 0.236 (0.029^*^) | -0.171 (0.087) | 0.054 (0.334) | -0.006 (0.481) | -0.168 (0.091) |
| CP_HC_ | -0.310 (0.058) | 0.367 (0.012^*^) | 0.252 (0.063) | 0.249 (0.066) | 0.320 (0.025^*^) | 0.234 (0.079) |
| ALPS | / | -0.077 (0.441) | -0.12 (0.228) | -0.028 (0.777) | -0.133 (0.179) | -0.010 (0.917) |
| ALPS_PD_ | / | 0.065 (0.605) | 0.048 (0.704) | -0.103 (0.413) | -0.171 (0.173) | 0.007 (0.959) |
| ALPS_HC_ | / | -0.086 (0.607) | -0.259 (0.116) | 0.191 (0.252) | 0.062 (0.709) | 0.028 (0.869) |
| **Right-hemisphere** | | | | | | |
| CP | -0.434 (0.001^***^) | 0.332 (0.001^***^) | 0.124 (0.107) | 0.163 (0.050) | 0.304 (0.001^**^) | 0.095 (0.170) |
| CP_PD_ | -0.357 (0.004^**^) | 0.233 (0.031^*^) | 0.008 (0.476) | 0.236 (0.029^*^) | 0.325 (0.004^**^) | -0.007 (0.477) |
| CP_HC_ | -0.512 (0.001^**^) | 0.373 (0.011^*^) | 0.096 (0.283) | 0.016 (0.462) | 0.004 (0.491) | 0.319 (0.025^*^) |
| ALPS | / | -0.148 (0.068) | -0.250 (0.005^**^) | -0.144 (0.073) | -0.301 (0.001^**^) | -0.147 (0.069) |
| ALPS_PD_ | / | -0.086 (0.248) | -0.057 (0.327) | -0.222 (0.038^*^) | -0.278 (0.012^*^) | -0.212 (0.045^*^) |
| ALPS_HC_ | / | -0.116 (0.245) | -0.466 (0.002^**^) | -0.010 (0.477) | -0.329 (0.022^*^) | 0.070 (0.337) |
| Results are presented as correlation coefficient (p-value).  Abbreviations: CP, ratio of choroid plexus volume/total intracranial volume; ALPS, diffusion tensor image analysis along the perivascular space; QSM, quantitative susceptibility mapping; SN, substantia nigra; RN, red nucleus; CN, caudate nucleus; PUT, putamen; GP, globus pallidus; _PD_, correlation analysis in Parkinson’s disease patients only; _HC_, correlation analysis in healthy controls only.  Significance levels: **p <* 0.05, ***p <* 0.01, ****p <* 0.001, Bonferroni-corrected. | | | | | | |

| **Supplementary Table 2** **Correlations between neuroimaging biomarkers and clinical characteristics in patients with Parkinson’s disease** | | | | | | | | | | | | |
| --- | --- | --- | --- | --- | --- | --- | --- | --- | --- | --- | --- | --- |
|  | **Age** | **Duration** | **H&Y** | **LEDD** | **UPDRS-Ⅱ** | **UPDRS-Ⅲ** | **FOG-Q** | **RBDSQ** | **MoCA** | **MMSE** | **HAM-D** | **HAM-A** |
| CP_L_ | 0.264 (0.017*) | -0.064 (0.307) | -0.104 (0.204) | -0.075 (0.276) | 0.064 (0.307) | 0.085 (0.250) | 0.229 (0.033^*^) | 0.238 (0.028^*^) | 0.018 (0.445) | 0.121 (0.169) | 0.117 (0.178) | 0.228 (0.034^*^) |
| CP_R_ | 0.487 (0.001***) | -0.005 (0.483) | -0.095 (0.227) | -0.097 (0.221) | 0.036 (0.388) | 0.164 (0.096) | 0.277 (0.013^*^) | 0.098 (0.219) | 0.048 (0.353) | -0.011 (0.466) | 0.108 (0.195) | 0.212 (0.045^*^) |
| ALPS_L_ | -0.396 (0.001^***^) | -0.078 (0.268) | -0.085 (0.251) | 0.195 (0.060) | -0.292 (0.009^**^) | -0.127 (0.157) | -0.243 (0.025^*^) | -0.037 (0.385) | -0.076 (0.274) | -0.138 (0.137) | -0.308 (0.006^**^) | -0.259 (0.019^*^) |
| ALPS_R_ | -0.438 (0.001^***^) | -0.207 (0.049^*^) | -0.085 (0.250) | 0.108 (0.196) | -0.269 (0.015^*^) | -0.265 (0.016^*^) | -0.280 (0.012^*^) | 0.039 (0.380) | -0.084 (0.252) | -0.006 (0.482) | -0.367 (0.001^***^) | -0.363 (0.001^***^) |
| SN_L_ | 0.043 (0.366) | -0.084 (0.253) | -0.064 (0.307) | 0.114 (0.182) | -0.046 (0.358) | 0.196 (0.059) | 0.057 (0.327) | 0.100 (0.214) | -0.053 (0.339) | -0.143 (0.127) | -0.193 (0.062) | -0.166 (0.093) |
| SN_R_ | 0.073 (0.282) | 0.089 (0.240) | 0.069 (0.293) | 0.198 (0.057) | 0.034 (0.393) | 0.309 (0.006^**^) | 0.169 (0.089) | -0.006 (0.480) | 0.071 (0.287) | -0.065 (0.304) | -0.167 (0.092) | -0.106 (0.200) |
| RN_L_ | -0.022 (0.432) | -0.087 (0.246) | -0.113 (0.186) | -0.016 (0.451) | -0.141 (0.131) | -0.102 (0.209) | -0.123 (0.164) | 0.018 (0.443) | 0.003 (0.490) | -0.05 (0.347) | -0.050 (0.347) | -0.049 (0.348) |
| RN_R_ | -0.039 (0.379) | -0.063 (0.308) | -0.076 (0.274) | -0.017 (0.448) | -0.091 (0.237) | -0.100 (0.215) | -0.069 (0.292) | -0.005 (0.484) | 0.034 (0.394) | -0.165 (0.094) | -0.113 (0.184) | -0.075 (0.277) |
| CN_L_ | 0.348 (0.002^**^) | 0.193 (0.062) | 0.084 (0.254) | 0.161 (0.101) | 0.136 (0.141) | 0.099 (0.217) | 0.213 (0.044^*^) | 0.087 (0.246) | 0.085 (0.252) | -0.272 (0.014^*^) | 0.293 (0.009^**^) | 0.301 (0.007^**^) |
| CN_R_ | 0.314 (0.005^**^) | 0.079 (0.265) | -0.039 (0.379) | 0.107 (0.198) | 0.039 (0.379) | -0.090 (0.238) | 0.078 (0.269) | 0.189 (0.066) | -0.01 (0.469) | -0.216 (0.042^*^) | 0.241 (0.027^*^) | 0.171 (0.087) |
| PUT_L_ | 0.483 (0.001^***^) | 0.215 (0.042^*^) | -0.048 (0.353^*^) | 0.045 (0.360) | 0.083 (0.257) | -0.012 (0.461) | 0.285 (0.011^*^) | 0.146 (0.123) | -0.092 (0.233) | -0.281 (0.012^*^) | 0.334 (0.003^**^) | 0.386 (0.001^***^) |
| PUT_R_ | 0.419 (0.001^***^) | 0.132 (0.148) | -0.060 (0.318) | 0.002 (0.495) | 0.041 (0.373) | -0.062 (0.312) | 0.263 (0.017^*^) | 0.082 (0.258) | -0.114 (0.182) | -0.271 (0.015^*^) | 0.234 (0.030^*^) | 0.303 (0.007^**^) |
| GP_L_ | 0.174 (0.082) | -0.067 (0.299) | -0.058（0.325） | 0.049 (0.348) | -0.208 (0.048^*^) | -0.095 (0.225) | 0.052 (0.341) | -0.04 (0.377) | -0.047 (0.355) | -0.034 (0.393) | -0.122 (0.166) | -0.145 (0.125) |
| GP_R_ | 0.235 (0.030^*^) | -0.018 (0.444) | 0.013 (0.460) | 0.030 (0.407) | -0.060 (0.317) | 0.001 (0.498) | 0.194 (0.061) | -0.024 (0.424) | -0.018 (0.445) | -0.062 (0.312) | -0.029 (0.409) | -0.033 (0.396) |
| Results are presented as correlation coefficient (*p*-value).  Abbreviations: CP, ratio of choroid plexus volume/total intracranial volume; ALPS, diffusion tensor image analysis along the perivascular space; SN, susceptibility value of substantia nigra; RN, susceptibility value of red nucleus; CN, susceptibility value of caudate nucleus; PUT, susceptibility value of putamen; GP, susceptibility value of globus pallidus; H&Y, Hoehn & Yahr scales; LEDD, levodopa equivalent daily dose; UPDRS, Unified Parkinson’s Disease Rating Scale; FOG-Q, Freezing of Gait Questionnaire; RBDSQ, REM Sleep Behaviour Disorder Screening Questionnaire; MoCA, Montreal Cognitive Assessment; MMSE, Mini-Mental State Examination; HAM-D, 17-item Hamilton Depression Rating Scale; HAM-A, Hamilton Anxiety Rating Scale; _L_, left-hemispheric; _R_, right-hemispheric.  Significance levels: **p <* 0.05, ***p <* 0.01, ****p <* 0.001, Bonferroni-corrected. | | | | | | | | | | | | |

**Supplementary Material 1**

The parameters of the three-dimensional T1-weighted magnetization prepared rapid acquisition gradient echo (3D-T1 MP-RAGE) sequence were as follows: repetition time (TR) = 2300 ms; echo time (TE) = 2.98 ms; inversion time (TI) = 900 ms; flip angle (FA) = 9°; slice thickness = 1 mm; field of view (FOV) = 256 × 256 × 192 mm³; matrix size = 256 × 256; voxel resolution = 1 × 1 × 1 mm³; and a total acquisition time (TA) of 5 min 30 s.

The parameters of the DTI sequence were: TR = 4100 ms; TE = 72 ms; FOV = 210 × 210 mm²; diffusion directions = 64; b-value = 1000 s/mm^2^; slice thickness = 2 mm; voxel size = 2 × 2 × 2 mm³; and a total TA of 5 min 18 s.

The parameters of the QSM sequence were: TR = 35ms; TE1 = 7.5 ms; TE2 = 14.42 ms; TE3 = 21.34 ms; TE4 = 28.26 ms; FA = 20°; slice thickness = 1 mm; FOV = 220 × 220 × 128 mm^3^; matrix size = 220 × 220 mm^2^; voxel resolution = 1 × 1 × 1 mm^3^; and a total TA of 7 min 21s.

**Supplementary Material 2**

**Choroid plexus volume calculation in linux**

recon-all -i input_t1.nii.gz -all -sd /output_dir -subjid subject1

**ALPS analysis in linux**

#!/bin/bash

## Mandatory inputs

SUBJECT_LIST=/media/sf_D_DRIVE/DTI_PDexercise/example_PD/PDlist

WORK_DIRS=/media/sf_D_DRIVE/DTI_PDexercise/example_PD

SCRIPT_DIRS=/media/sf_D_DRIVE/matlab2016b-setup-software/DTIscript/DTI

## Optional inputs

#DICOM inputs

T1_DICOM_DIRS=/media/sf_D_DRIVE/DTI_PDexercise/T1_PD

DTI_DICOM_DIRS=/media/sf_D_DRIVE/DTI_PDexercise/DTI_PD

gradT=-iz

DoBedpostx=no

nthread=27

## default parameters

FDTpref=fdt

DTKpref=dtk

T1brainTemplate=$SCRIPT_DIRS/MNI152_T1_2mm_brain.nii.gz

T1Template=$SCRIPT_DIRS/MNI152_T1_2mm.nii.gz

logdir=$WORK_DIRS/logs

T1_DIR=$WORK_DIRS/T1

DTI_DIR=$WORK_DIRS/DTI

#### functions ##########

pipe(){

T1_SUB_DIR=$1

DTI_SUB_DIR=$2

logpath=$3

run echo logpath=$logpath >> $logpath

## DICOM2NII

# dicom2nii T1

if [ -n "$T1_DICOM_DIRS" ]

then run echo " --DICOM2NII T1: $s"

if [ ! -f $T1_SUB_DIR/T1.nii ]

then

run dcm2niix -f T1 -o $T1_SUB_DIR/ -z no $T1_DICOM_DIRS/$s >> $logpath

fi

fi

# dicom2nii DTI

if [ -n "$DTI_DICOM_DIRS" ]

then run echo " --DICOM2NII DTI: $s"

if [ ! -f $DTI_SUB_DIR/dti.nii.gz ]

then

run dcm2niix -f dti -z y -o $DTI_SUB_DIR/ $DTI_DICOM_DIRS/$s >> $logpath

run mv -f $DTI_SUB_DIR/dti.bval $DTI_SUB_DIR/bvals >> $logpath

run mv -f $DTI_SUB_DIR/dti.bvec $DTI_SUB_DIR/bvecs >> $logpath

fi

fi

## eddycurrent and motion correction

run echo " --EddyCurrent and Motion Correction: $s"

if [ ! -f $DTI_SUB_DIR/data.nii.gz ]

then

run eddy_correct $DTI_SUB_DIR/dti $DTI_SUB_DIR/data 0 >> $logpath

fi

##Brain Extract

# bet DTI

run echo " --BrainExtract DTI: $s"

if [ ! -f $DTI_SUB_DIR/nodif_brain_mask.nii.gz ]

then

run fslroi $DTI_SUB_DIR/data $DTI_SUB_DIR/b0 0 1 >> $logpath

run bet $DTI_SUB_DIR/b0 $DTI_SUB_DIR/nodif_brain -m -f 0.2 -R >> $logpath

fi

#bet T1

run echo " --BrainExtract T1: $s"

if [ ! -f $T1_SUB_DIR/T1_brain.nii ]

then

run bet $T1_SUB_DIR/T1.nii $T1_SUB_DIR/T1_brain -m -f 0.5 -R -S -B >> $logpath

run gunzip $T1_SUB_DIR/T1_brain.nii.gz >> $logpath

rm -f $T1_SUB_DIR/T1_brain.nii.gz >> $logpath

fi

#DTI fit

run echo " --DTI FIT based on FDT: $s"

if [ ! -f $DTI_SUB_DIR/${FDTpref}_FA.nii.gz ]

then

run dtifit -k $DTI_SUB_DIR/data -o $DTI_SUB_DIR/$FDTpref -m $DTI_SUB_DIR/nodif_brain_mask -r $DTI_SUB_DIR/bvecs -b $DTI_SUB_DIR/bvals >> $logpath

run fslmaths $DTI_SUB_DIR/${FDTpref}_FA -nan -min 1 -ero -bin ${DTI_SUB_DIR}/brainmask >> $logpath

for m in FA L1 L2 L3 MD MO

do

run fslmaths $DTI_SUB_DIR/${FDTpref}_${m} -mul $DTI_SUB_DIR/brainmask $DTI_SUB_DIR/${FDTpref}_clean_${m} >> $logpath

done

run fslmaths $DTI_SUB_DIR/${FDTpref}_clean_L1 -add $DTI_SUB_DIR/${FDTpref}_clean_L2 -div 2 $DTI_SUB_DIR/${FDTpref}_clean_L23 >> $logpath

fi

# DTK recon

run echo " --DTK recon: $s "

if [ ! -f $DTI_SUB_DIR/${DTKpref}_tensor.nii.gz ]

then

# gradient

if [ ! -f $DTI_SUB_DIR/dtk_dgt ]

then run transpose $DTI_SUB_DIR/bvecs $DTI_SUB_DIR/bvecsT >> $logpath

run transpose $DTI_SUB_DIR/bvals $DTI_SUB_DIR/bvalsT >> $logpath

awk '{print NR,$1}' ${DTI_SUB_DIR}/bvalsT >${DTI_SUB_DIR}/tmp2

awk '{print NR,$1,$2,$3}' ${DTI_SUB_DIR}/bvecsT >${DTI_SUB_DIR}/tmp1

join ${DTI_SUB_DIR}/tmp1 ${DTI_SUB_DIR}/tmp2 |awk '{print $2,$3,$4,$5}' > ${DTI_SUB_DIR}/tmp

grep -v '^\s*$' ${DTI_SUB_DIR}/tmp >${DTI_SUB_DIR}/tmp1

sed 's/ /,/g' ${DTI_SUB_DIR}/tmp1 >${DTI_SUB_DIR}/dtk_dgt

rm -f $DTI_SUB_DIR/bv*T $DTI_SUB_DIR/tmp*

fi

#dti_recon

if [ ! -f $DTI_SUB_DIR/data_brain.nii.gz ]

then

run fslmaths $DTI_SUB_DIR/data -mas $DTI_SUB_DIR/brainmask $DTI_SUB_DIR/data_brain >> $logpath

fi

run dti_recon $DTI_SUB_DIR/data_brain.nii.gz ${DTI_SUB_DIR}/$DTKpref -gm ${DTI_SUB_DIR}/dtk_dgt -ot nii.gz >> $logpath

fi

## Deterministic fiber tracking

run echo " --Deterministic fiber tracking: $s"

if [ ! -f $DTI_SUB_DIR/${DTKpref}.trk ]

then

run dti_tracker ${DTI_SUB_DIR}/$DTKpref ${DTI_SUB_DIR}/${DTKpref}.trk -at 80 $gradT -m ${DTI_SUB_DIR}/${FDTpref}_clean_FA.nii.gz 0.1 1 -it nii.gz >> $logpath

spline_filter ${DTI_SUB_DIR}/${DTKpref}.trk 1 ${DTI_SUB_DIR}/${DTKpref}S.trk >> $logpath

fi

## Probabilistic fiber tracking

if [ "$DoBedpostx" == "yes" ]

then run echo " --Bedpostx(for Probabilistic fiber tracking): $s"

if [ ! -f ${DTI_SUB_DIR}/bedpostX/merged_f1samples.nii.gz ]

then

run bedpostx $DTI_SUB_DIR >> $logpath

mv ${DTI_SUB_DIR}.bedpostX ${DTI_SUB_DIR}/bedpostX >> $logpath

fi

fi

##Spatial normalization

run echo " --Spatial Normalization using linear (diff2T1) + nonlinear (T12MNI) method: $s"

if [ ! -f $DTI_SUB_DIR/diff2str.mat ]

then

run flirt -in $DTI_SUB_DIR/nodif_brain -ref $T1_SUB_DIR/T1_brain -omat $DTI_SUB_DIR/diff2str.mat -o $DTI_SUB_DIR/nodif_brain_2_T1_linear >> $logpath

fi

if [ ! -f $T1_SUB_DIR/str2standard.mat ]

then

run flirt -in $T1_SUB_DIR/T1_brain -ref $T1brainTemplate -omat $T1_SUB_DIR/str2standard.mat -o T1_brain_2_MNI_linear >> $logpath

fi

if [ ! -f $DTI_SUB_DIR/diff2standard.mat ]

then

run convert_xfm -omat $DTI_SUB_DIR/diff2standard.mat -concat $T1_SUB_DIR/str2standard.mat $DTI_SUB_DIR/diff2str.mat >> $logpath

run convert_xfm -omat $DTI_SUB_DIR/str2diff.mat -inverse $DTI_SUB_DIR/diff2str.mat >> $logpath

run convert_xfm -omat $DTI_SUB_DIR/standard2diff.mat -inverse $DTI_SUB_DIR/diff2standard.mat >> $logpath

fi

if [ ! -f $T1_SUB_DIR/str2standard_warp.nii.gz ]

then

run fnirt --in=$T1_SUB_DIR/T1 --aff=$T1_SUB_DIR/str2standard.mat --cout=$T1_SUB_DIR/str2standard_warp --config=T1_2_MNI152_2mm --ref=$T1Template >> $logpath

run invwarp -w $T1_SUB_DIR/str2standard_warp -o $T1_SUB_DIR/str2standard_invwarp -r $T1_SUB_DIR/T1 >> $logpath

fi

if [ ! -f $DTI_SUB_DIR/str2standard_warp.nii.gz ]

then

run cp $T1_SUB_DIR/str2standard_*warp.nii.gz $DTI_SUB_DIR/

fi

if [ ! -f $DTI_SUB_DIR/MNI_${FDTpref}_clean_FA.nii.gz ]

then

applywarp --in=$DTI_SUB_DIR/nodif_brain --ref=$T1Template --warp=$T1_SUB_DIR/str2standard_warp --premat=$DTI_SUB_DIR/diff2str.mat --out=$DTI_SUB_DIR/MNI_nodif_brain >> $logpath

applywarp --in=$T1_SUB_DIR/T1_brain --ref=$T1Template --warp=$T1_SUB_DIR/str2standard_warp --out=$T1_SUB_DIR/MNI_T1_brain >> $logpath

for m in FA MD L1 L2 L23 MO

do

run applywarp --in=$DTI_SUB_DIR/${FDTpref}_clean_$m --ref=$T1Template --warp=$T1_SUB_DIR/str2standard_warp --premat=$DTI_SUB_DIR/diff2str.mat --out=$DTI_SUB_DIR/MNI_${FDTpref}_clean_$m >> $logpath

done

fi

run echo "====Finish DTI Processing for Subject: $s====" >> $logpath

}

run()

{

f="$@"

echo -----------------------------------------

if [ "${f:0:4}" = "echo" ]

then

$f

else

echo $f

$f

fi

}

##### main code #####

export PATH=${SCRIPT_DIRS}:$PATH

dtkpath=`which dtk`

DSI_PATH=${dtkpath%/*}/matrices

export DSI_PATH

if [ ! -d $logdir ];then mkdir $logdir -p;fi

if [ ! -d $T1_DIR ];then mkdir $T1_DIR -p;fi

if [ ! -d $DTI_DIR ];then mkdir $DTI_DIR -p;fi

k=1

for s in `cat $SUBJECT_LIST`

do run echo "====Start DTI Processing for Subject: $s===="

T1_SUB_DIR=$T1_DIR/$s

DTI_SUB_DIR=$DTI_DIR/$s

logpath=$logdir/dti_${s}.txt

export logpath

if [ ! -d $T1_SUB_DIR ];then mkdir $T1_SUB_DIR;fi

if [ ! -d $DTI_SUB_DIR ];then mkdir $DTI_SUB_DIR;fi

pipe $T1_SUB_DIR $DTI_SUB_DIR $logpath &

if [ $((k%nthread)) -eq 0 ]

then

echo --------wraiting for job $k - $s finish

wait

fi

k=$((k+1))

Done

fslmaths dwi.nii.gz -mas wm_mask.nii.gz -Tmean dwi_wm_mean.nii.gz && fslmaths dwi.nii.gz -mas csf_mask.nii.gz -Tmean dwi_csf_mean.nii.gz && fslstats dwi_wm_mean.nii.gz -M | awk '{print "ALPS index: " $1}' > alps_result.txt

**QSM Registration in MATLAB**

spm('defaults', 'FMRI');

spm_jobman('initcfg');

base_dir = 'F:\QSM_data_APART';

output_dir = fullfile(base_dir, 'Normalised_Results');

if ~exist(output_dir, 'dir')

mkdir(output_dir);

end

subj_dirs = dir(fullfile(base_dir, 'PD*'));

subj_dirs = subj_dirs([subj_dirs.isdir]);

subj_ids = {subj_dirs.name};

for subj_idx = 1:length(subj_ids)

subj_id = subj_ids{subj_idx};

fprintf('\nProcessing subject: %s (%d/%d)\n', subj_id, subj_idx, length(subj_ids));

subj_dir = fullfile(base_dir, subj_id, 'results');

required_files = {[subj_id '_T1.nii'], [subj_id '_imag.nii'], [subj_id '_QSM_STAR.nii'], [subj_id '_mask.nii']};

missing_files = {};

for f = 1:length(required_files)

if ~exist(fullfile(subj_dir, required_files{f}), 'file')

missing_files{end+1} = required_files{f};

end

end

if ~isempty(missing_files)

fprintf('Skipping subject %s - Missing files: %s\n', subj_id, strjoin(missing_files, ', '));

continue;

end

matlabbatch = {};

matlabbatch{1}.spm.spatial.coreg.estwrite.ref = {fullfile(subj_dir, [subj_id '_T1.nii'])};

matlabbatch{1}.spm.spatial.coreg.estwrite.source = {fullfile(subj_dir, [subj_id '_imag.nii'])};

matlabbatch{1}.spm.spatial.coreg.estwrite.other = {fullfile(subj_dir, [subj_id '_QSM_STAR.nii'])};

matlabbatch{1}.spm.spatial.coreg.estwrite.eoptions.cost_fun = 'nmi';

matlabbatch{1}.spm.spatial.coreg.estwrite.eoptions.sep = [4 2];

matlabbatch{1}.spm.spatial.coreg.estwrite.eoptions.tol = [0.02 0.02 0.02 0.001 0.001 0.001 0.01 0.01 0.01 0.001 0.001 0.001];

matlabbatch{1}.spm.spatial.coreg.estwrite.eoptions.fwhm = [7 7];

matlabbatch{1}.spm.spatial.coreg.estwrite.roptions.interp = 4;

matlabbatch{1}.spm.spatial.coreg.estwrite.roptions.wrap = [0 0 0];

matlabbatch{1}.spm.spatial.coreg.estwrite.roptions.mask = 0;

matlabbatch{1}.spm.spatial.coreg.estwrite.roptions.prefix = 'r';

matlabbatch{2}.spm.spatial.normalise.estwrite.subj.vol = {fullfile(subj_dir, [subj_id '_T1.nii'])};

matlabbatch{2}.spm.spatial.normalise.estwrite.subj.resample(1) = cfg_dep('Coregister: Estimate & Reslice: Resliced Images', substruct('.','val', '{}',{1}, '.','val', '{}',{1}, '.','val', '{}',{1}, '.','val', '{}',{1}), substruct('.','rfiles'));

matlabbatch{2}.spm.spatial.normalise.estwrite.eoptions.biasreg = 0.0001;

matlabbatch{2}.spm.spatial.normalise.estwrite.eoptions.biasfwhm = 60;

matlabbatch{2}.spm.spatial.normalise.estwrite.eoptions.tpm = {'F:\Program Files\MATLAB\R2024b\toolbox\spm12\spm12\tpm\TPM.nii'};

matlabbatch{2}.spm.spatial.normalise.estwrite.eoptions.affreg = 'mni';

matlabbatch{2}.spm.spatial.normalise.estwrite.eoptions.reg = [0 0.001 0.5 0.05 0.2];

matlabbatch{2}.spm.spatial.normalise.estwrite.eoptions.fwhm = 0;

matlabbatch{2}.spm.spatial.normalise.estwrite.eoptions.samp = 3;

matlabbatch{2}.spm.spatial.normalise.estwrite.woptions.bb = [-91 -126 -72; 90 91 109];

matlabbatch{2}.spm.spatial.normalise.estwrite.woptions.vox = [1 1 1];

matlabbatch{2}.spm.spatial.normalise.estwrite.woptions.interp = 4;

matlabbatch{2}.spm.spatial.normalise.estwrite.woptions.prefix = 'w';

matlabbatch{3}.spm.spatial.coreg.estwrite.ref = {fullfile(subj_dir, [subj_id '_T1.nii,1'])};

matlabbatch{3}.spm.spatial.coreg.estwrite.source = {fullfile(subj_dir, [subj_id '_imag.nii,1'])};

matlabbatch{3}.spm.spatial.coreg.estwrite.other = {fullfile(subj_dir, [subj_id '_mask.nii,1'])};

matlabbatch{3}.spm.spatial.coreg.estwrite.eoptions.cost_fun = 'nmi';

matlabbatch{3}.spm.spatial.coreg.estwrite.eoptions.sep = [4 2];

matlabbatch{3}.spm.spatial.coreg.estwrite.eoptions.tol = [0.02 0.02 0.02 0.001 0.001 0.001 0.01 0.01 0.01 0.001 0.001 0.001];

matlabbatch{3}.spm.spatial.coreg.estwrite.eoptions.fwhm = [7 7];

matlabbatch{3}.spm.spatial.coreg.estwrite.roptions.interp = 0;

matlabbatch{3}.spm.spatial.coreg.estwrite.roptions.wrap = [0 0 0];

matlabbatch{3}.spm.spatial.coreg.estwrite.roptions.mask = 0;

matlabbatch{3}.spm.spatial.coreg.estwrite.roptions.prefix = 'r';

matlabbatch{4}.spm.spatial.normalise.estwrite.subj.vol = {fullfile(subj_dir, [subj_id '_T1.nii,1'])};

matlabbatch{4}.spm.spatial.normalise.estwrite.subj.resample(1) = cfg_dep('Coregister: Estimate & Reslice: Resliced Images', substruct('.','val', '{}',{3}, '.','val', '{}',{1}, '.','val', '{}',{1}, '.','val', '{}',{1}), substruct('.','rfiles'));

matlabbatch{4}.spm.spatial.normalise.estwrite.eoptions.biasreg = 0.0001;

matlabbatch{4}.spm.spatial.normalise.estwrite.eoptions.biasfwhm = 60;

matlabbatch{4}.spm.spatial.normalise.estwrite.eoptions.tpm = {'F:\Program Files\MATLAB\R2024b\toolbox\spm12\spm12\tpm\TPM.nii'};

matlabbatch{4}.spm.spatial.normalise.estwrite.eoptions.affreg = 'mni';

matlabbatch{4}.spm.spatial.normalise.estwrite.eoptions.reg = [0 0.001 0.5 0.05 0.2];

matlabbatch{4}.spm.spatial.normalise.estwrite.eoptions.fwhm = 0;

matlabbatch{4}.spm.spatial.normalise.estwrite.eoptions.samp = 3;

matlabbatch{4}.spm.spatial.normalise.estwrite.woptions.bb = [-91 -126 -72; 90 91 109];

matlabbatch{4}.spm.spatial.normalise.estwrite.woptions.vox = [1 1 1];

matlabbatch{4}.spm.spatial.normalise.estwrite.woptions.interp = 0;

matlabbatch{4}.spm.spatial.normalise.estwrite.woptions.prefix = 'w';

job_file = fullfile(output_dir, [subj_id '_QSM_Normalise_job.mat']);

save(job_file, 'matlabbatch');

try

spm_jobman('run', matlabbatch);

fprintf('Successfully processed subject: %s\n', subj_id);

catch ME

fprintf('Error processing subject %s: %s\n', subj_id, ME.message);

end

end

fprintf('\nAll subjects processed!\n');
